# Annotations

**Published:** 1901-12-14

**Authors:** 


### ANNOTATIONS.

### The Incubus of the London University.

The teachers at the Metropolitan Medical Schools have recently been somewhat unduly awakened to the fact that London is losing its popularity as a centre of medical education. It has been shown that instead of coming to London the tendency of students is to drift towards Scottish and Provincial Universities, and, although various explanations of the fact have been given, one can hardly doubt that the chief reason which actuates them in so doing is the knowledge that at these places the average student can get a degree, while in London the degree is reserved for those who may properly be regarded as honours men. This is at the bottom of the whole business, and no one can doubt that if the London University were managed in the interest of London, its inhabitants and its teaching institutions, this condition of affairs would be rectified to-morrow. A speaker at the Authors' Club said last Monday that the University had before it the possibility of fizzling off to nothing or becoming one of the most important agencies for the development of London—a statement which will doubtless be accepted with different meanings by the disputants on the two sides. Unfortunately the University, although London by name, has all these years been managed in the interests of the world at large, and even now, notwithstanding its new constitution and all the hopes that have been raised by it, there seems but little probability of any such changes being made as to draw the stream of medical students back again to London. What is wanted in order to render London attractive as a place of medical study is that every student should be able to feel that if he works steadily and well he may obtain a degree at the end of his course. By all means let the University of London do what it likes in the way of making its "first-class" and its gold medals difficult of access, but let it give a pass degree to the steady student of average power, as other Universities do. We have no sympathy with the attempts which are being made to induce the colleges to "give degrees" to their diplomates, nor do we think that such degrees would be worth much when they were given. But the very fact that such schemes as we hear of now and again for providing practitioners with degrees are being seriously discussed, shows how keenly the difficulty is being felt.

### The Difficulty at Macclesfield.

The deadlock at the Macclesfield Infirmary continues. The governors having appointed a lady as junior house surgeon, have now asked her to resign, offering her a year's salary. The lady, however, has declined to do so, saying that just as the medical men asserted that this was a question of principle with them, so it was a question of principle with her, and that she was fighting the battle of medical women, etc. All of which shows how easy it is to muddle up different questions. The question of principle with the medical staff, as we understand it, is whether they or the Board shall select the house surgeon. The sex of the house surgeon is another affair. Certainly, so far as the profession at large is concerned, there is no principle involved in the employment or the non-employment of women except that of doing the best for the patients. We know that young lady enthusiasts hold to the motto of the Garter, *Honi soit qui mal y pense*, and say that all is pure in science. In this, surely, we may agree. Nevertheless, we think that those who are responsible for the conduct of a hospital are not doing right in arranging beforehand that difficulties and complications shall arise in the management of the institution. Why should these problems always be fought out over hospital patients? If it can be shown that the things we have in our minds are done by medical women in private practice, then *cadit quæstio*.

### Following Midwives.

A very serious question is raised by the action of certain medical men in refusing to "follow midwives," *i.e.*, to attend confinements, in dealing with which the midwives in charge have found themselves in difficulties. At several inquests during the past few months, it has come out that medical men have declined to attend, not because they have been busy or indisposed, and in some cases not even because it was a question of fee, but definitely because they declined to "follow midwives." Now, although it is quite clear that a practitioner may always please himself as to whether he attends a case or not, the reason these doctors gave for their action seems to us peculiarly unfortunate in the present position of the midwives question. Everyone, we imagine, will admit that normal child-birth is an ordinary function of woman, and that all that is required in the attendant, *i.e.* the midwife, is a careful training in the very narrow range of work which she will have to perform, and strict obedience to the rules laid down for her guidance. There is nothing medical about it at all. Child-birth, however, like every other function of the body, is liable to go wrong, and the treatment of abnormal child-birth, just the same as the treatment of any other derangement, is the work of the doctor. Hence, what is wanted is that every parturient woman should be actually attended by a properly trained midwife, and at the same time have the assurance that if things should go wrong medical aid shall be available. Under such an arrangement the training of the midwife need never be more than elementary, she never need be an expensive person, and midwives capable of doing all that is necessary might exist in every hamlet and be within the reach of every paturient woman. Such midwives would not be "competitors" in the field of medicine, nor would they be in any sense a "new and inferior order of medical practitioners." When, however, we find qualified practitioners refusing to "follow midwives," the whole aspect of the case is altered, and we are not surprised to find that those who aim at a much more advanced training and a much higher position for the midwife than is suggested above, should seize the opportunity and should endeavour to show that in face of such a "boycott" the only safe course is to give the midwives such a training as shall enable them to conduct cases even of difficult labour. The claim of the highly-skilled yet medically-ignorant midwife to treat cases which are abnormal is one greatly to be deprecated. Yet it is a claim which will be very difficult to resist if the refusal to "follow midwives" should become common among qualified practitioners.

